# Influence of Sleep Disturbances on Quality of Life of Iranian Menopausal Women

**DOI:** 10.1155/2013/907068

**Published:** 2013-11-05

**Authors:** Zohreh Yazdi, Khosro Sadeghniiat-Haghighi, Amir Ziaee, Khadijeh Elmizadeh, Masomeh Ziaeeha

**Affiliations:** ^1^Occupational Medicine, Qazvin University of Medical Sciences, Qazvin, Iran; ^2^Occupational Sleep Research Center, Tehran University of Medical Sciences, Tehran, Iran; ^3^Metabolic Disease Research Center, Qazvin University of Medical Sciences, Qazvin, Iran; ^4^Qazvin University of Medical Sciences, Bahonar Bolvar, P.O. Box 3419759811, Qazvin, Iran

## Abstract

*Background*. Subjective sleep disturbances increase during menopause. Some problems commonly encountered during menopause, such as hot flushes and sweating at night, can cause women to have difficulty in sleeping. These complaints can influence quality of life of menopausal women. *Methods*. This cross-sectional study was performed on menopausal women attending health centers in Qazvin for periodic assessments. We measured excessive daytime sleepiness by Epworth sleepiness scale (ESS), obstructive sleep apnea (OSA) by the Berlin questionnaire, and insomnia by the insomnia severity index (ISI). We evaluate quality of life by the Menopause specific quality of life questionnaire (MENQOL). *Results*. A total of 380 menopausal women entered the study. Mean age of participated women was 57.6 ± 6.02. Mean duration of menopause was 6.3 ± 4.6. The frequency of severe and moderate insomnia was 8.4% (32) and 11.8% (45). Severe daytime sleepiness (ESS ≥ 10) was present in 27.9% (80) of the participants. Multivariate analytic results show that insomnia and daytime sleepiness have independent negative impact on each domain and total score of MENQOL questionnaire. *Conclusion*. According to our findings, EDS and insomnia are frequent in menopausal women. Both EDS and insomnia have significant quality of life impairment.

## 1. Introduction

Across the lifespan, sleep disturbances are more prevalent in women than men.

Pregnancy, menstrual-related fluctuations, and the menopause may cause high prevalence of sleep disturbances in women [1–3]. Sleep disorders are more common during the menopausal transition as compared to premenopausal years [4]. During the menopause, progressive decline in estrogen and progesterone levels are frequently related to vasomotor symptoms, night sweats, fatigue, mood alterations, irritability, headache, palpitation, and sleep disorders [5, 6]. These symptoms may be subtle to severe and disabling. Sleeping disturbances are among the most frequent health complaints of women during menopause. They include disorders such as difficulty of falling asleep, fractioned sleep, night-time awakening, inability of resuming sleep, problems in waking up, fatigue, and daytime sleepiness [7, 8]. 

Insomnia is a common disorder in menopausal women with the prevalence of 28–63% based on different studies [9]. Many women, during the menopause, achieve less than 6 h of sleep on a regular basis, becoming at higher risk for short-term and long-term problems. The main effects of short-term sleep deprivation include anxiety, drowsiness, memory and cognitive impairment, and stressed relationship. Also, the most important long-term effects of sleep deprivation include high blood pressure, heart attack, stroke, and depression, and other mood disorders. Overall, insomnia is a more common disorder among the women than the men, and those with preexisting chronic insomnia may have increased vulnerability for an exacerbation of sleep disturbance during menopause [10, 11]. 

Studies have shown that the prevalence and severity of sleep apnea increases during menopause. Menopausal women are prone to higher prevalence of obstructive sleep apnea due to weight gain and decrease in estrogen and progesterone levels [12, 13]. 

A study on 589 middle aged women showed that mild obstructive sleep apnea was 2.6 times and severe obstructive sleep apnea was 3.5 times more prevalent in postmenopausal than in premenopausal women [14]. 

Fatigue and daytime sleepiness are other symptoms of sleep disorders in premenopausal women [15]. In one study, 149 women aged 40–59 years were evaluated regarding daytime sleepiness. They showed the prevalence of daytime sleepiness to be 33.6%. Postmenopausal status, sedentarism, and hot flush were risk factors for increased daytime sleepiness [16]. Insomnia and obstructive sleep apnea can also increase daytime sleepiness. Several studies on different populations have shown that sleep quality is an important variable in overall health and quality of life [1, 4, 17]. Investigations on the patients with epilepsy, end stage cancer, and obstructive pulmonary disease have shown that sleep disorders can decrease the quality of life [18–20]. In one study in 2009, the correlation between different sleep disorders and quality of life was investigated in 887 women aged 45–59 years. They showed a prevalence of 54% for sleep disorders and women with different sleep disorders had lower quality of life regarding vasomotor, psychosocial, physical, and sexual aspects [21]. 

In different studies in Iran, high prevalence of sleep disorders in menopausal women has been shown. One study showed a prevalence of 70% for sleep disorders in women aged 50–60 years [22, 23]. However, there is no study regarding the effect of sleep disorders on quality of life in menopausal women in Iran. 

This study was performed to evaluate the prevalence of insomnia, daytime sleepiness, and obstructive sleep apnea in menopausal women. We also evaluated the effect of these disorders on quality of life. 

## 2. Methods and Materials 

This cross-sectional study was performed on menopausal women attending health centers in Qazvin for periodic assessments. In a three-month period, all women aged 50–60 years with a history of at least one year of amenorrhea entered the study. Patients with a history of hysterectomy, premature menopause, hormonal replacement therapy, diabetes mellitus, hypertension, hypnotic drug consumption, and heart failure were excluded from the study. All women filled out a questionnaire containing demographic data, age, years of menopause, family income, and marital state. Height and weight of all cases were measured. 

Insomnia severity index (ISI), Epworth sleepiness scale (ESS), and Berlin questionnaire (BQ) were used to assess sleep disorders. Menopause specific quality of life questionnaire (MENQOL) was used to evaluate the quality of life. 

The Persian translation of all questionnaires used in the study was confirmed to be valid in previous studies. The Cronbach's Alpha values in the previous studies conducted in the Persian language were 0.81, 0.89, 0.79, and 0.77, respectively [24–27]. 

The insomnia severity index is composed of seven items measuring the symptoms and consequences of insomnia. The perceived severity of each item is rated on a 0–4 Likert scale ranging from 0 (not at all) to 4 (very severe). A total score “8” or greater indicates insomnia [24].

The Epworth Sleepiness Scale consist eight questions that subjectively evaluate urge of patients to sleep of different situations in life. ESS was designed based on Likert scale rating from 0 to 3. Scores ≥10 are considered excessive daytime sleepiness [25]. 

The BQ includes eight items about snoring, daytime somnolence, and history of hypertension. The patients were categorized as being at a low risk or high risk of having sleep apnea [26]. 

The MENQOL is a 29-item questionnaire that assesses the effects of the menopausal symptoms, divided into 4 categories, physical (16 items), vasomotor (3 items), psychological (7 items), and sexual (3 items) on quality of life in postmenopausal women. The likert scale with seven points scoring system used during the administration of questionnaire. For all items, this seven-point likert scale is converted to an eight-point scale, ranging from 1 (no experience of menopausal symptom) to 8 (the most severe symptom). The higher the scores are considered to be the worse the quality of life [27]. 

In our samples, Cronbach's alpha values for these questionnaires were found to be 0.82, 0.78, 0.88, and 0.83, respectively. 

The questionnaires were completed by a trained person and the participants were requested to answer the questions carefully. 

 All participants were informed about the research and written consent obtained. 

Data analysis was performed using SPSS software version 13. Mean and SD scores were calculated for numerical variables and number and percent for categorical variables. We performed multiple regression analysis between dependent variable (insomnia, EDS, and OSA) and independent variables (age, duration of menopause, income, marital status, and BMI). We also performed multiple regression analysis between subscales of quality of life and insomnia, EDS and OSA as independent variables. *P* value <0.05 was considered statistically significant. 

## 3. Results

A total of 380 menopausal women entered the study. Demographic data is shown in Table 1. The mean age of the participants was 57.6 (6.02) years with the maximum and minimum of 77 and 46, respectively. The mean duration of menopause was 6.3 (4.6) years with the maximum and minimum of 25 and 1, respectively. The mean duration of night sleep was 7.6 (5.2) hours. The results of the answers to each part of the MENQOL questionnaire was 3.9 (1.9), 17.4 (7.1), 32.5 (11.9), and 3.1 (1.3) in the subscales of vasomotor, psychosocial, physical, and sexual aspects, respectively. The frequency of severe and moderate insomnia was 8.4% (32) and 11.8% (45). Daytime sleepiness (ESS ≥ 10) was present in 27.9% (80) of the participants.

As shown in Table 2, insomnia has a strong statistical correlation with age and duration of menopause. With increasing age and duration of menopause, the frequency of insomnia increases. In regard to daytime sleepiness, there was only relation between daytime sleepiness and BMI. It is also shown that the frequency of OSA increases with increasing BMI. 

Table 3 shows that menopausal women with daytime sleepiness or insomnia have lower scores in all domains of menopausal-specific quality of life questionnaire and also in total score. On the other hand, women with the diagnosis of obstructive sleep apnea based on Berlin questionnaire had lower scores in the psychosocial domain of the questionnaire. 

Multivariate analytic results (Table 4) show that insomnia and daytime sleepiness have independent negative impact on each domain and total score of MENQOL questionnaire. However, obstructive sleep apnea has negative impact only on psychosocial domain of the questionnaire. 

## 4. Discussion 

The results of the present study showed that the frequency of insomnia and daytime sleepiness in participating women were higher than in the general population. It was also shown that both insomnia and daytime sleepiness had independently negative impact on all aspects of quality of life but OSA had negative impact only on psychosocial domain of questionnaire. 

In regard to high prevalence of sleep disorders in menopausal women, different studies have been performed to determine the causative factors. In one cross-sectional study, the prevalence of daytime sleepiness and its risk factors was evaluated in 149 women aged 40–59 years. The authors used Epworth questionnaire to assess daytime sleepiness. They reported that 33.6% of the participants had daytime sleepiness. They showed that sedentarism and hot flush were the most important risk factors for increased daytime sleepiness [16]. We showed a correlation between increased BMI and sleepiness. This correlates with the findings of other studies because sedentarism leads to increased BMI [16, 23]. 

 In present study, the prevalence of moderate to severe insomnia was 20% that was higher in contrast to a similar study [10]. Increasing age and duration of menopause were the most important risk factors of insomnia and this correlates well with the results of the bulk of studies. 

In one study regarding the risk factors of insomnia in menopausal women, the authors showed that the severity of hot flash and sedentarism had a role in insomnia but there was no link between age and insomnia [10]. The different results can be explained by the difference between the age ranges of the study population. The median age in our study was 58 but it was 48 in their series. However, the mean sleep time in our series was shown to be equal to the proposed time for sleep. 

Studies using polysomnography showed that menopausal women did not have lower sleep time than the others. They had lower sleep quality. Also, in other studies, the women had a trend to have a higher amount of sleep time [1, 28]. 

Sleep disturbances during menopause are often attributed to nocturnal hot flashes and sweats associated with changing hormone patterns. Hot flashes and night sweating can disrupt sleep. Estrogen leads to shorter sleep latency and increased number and duration of REM phase of sleep. Decreased estrogen level causes longer sleep latency and shorter REM sleep leading to tiredness after awaking [12, 29]. 

The results of the present study showed that menopausal women had lower quality of life and the hot flash was the most common complain. The results of other studies showed that vasomotor symptoms during menopause can lead to stress and sleep disorders [21]. 

In this study we showed the negative impact of sleep disorders especially insomnia and daytime sleepiness on quality of life. Other studies on different populations using different questionnaires also showed similar results [1, 10, 16].

One study evaluated 2800 males and females in the age range of 53–97 years. The authors showed that sleep disorders especially insomnia are related to lower quality of life. They used SF-36 questionnaire for quality of life assessment [30]. Another study comparing patients with insomnia with healthy controls showed that insomnia patients had markedly lower scores in all domains of quality of life. 

As referred to study results, we can conclude that insomnia and daytime sleepiness can lead to somatic pains, impaired social relationships, and psychosocial and sexual problems. We also found a relation between OSA and psychosocial domain. Several studies have shown the negative impact of OSA on memory and concentration.

Finally, as for the limitations of the present study one can mention its cross-sectional design. As in this study, the factors of age and duration of menopause had effect on insomnia; we suggest a cohort study to be designed for long term evaluation of women. The other limitation of the study was the subjective evaluation of sleep disorders. Using objective methods like polysomnography and actigraphy can lead to more strict results in future studies. The point of power in this study was its relatively big study population which could reflect the real condition in the society. 

## 5. Conclusion 

As regards the results of this study, a normal sleep is an important factor for general health. Quality of life includes psychosomatic, social, and physical aspects and sleep disorders can impair all of them. We can conclude that in periodic assessments of menopausal women, it is necessary to concentrate on diagnosis and treatment of sleep disorders. In addition evaluation of effectiveness of different treatment methods is necessary in this group. It is also recommended to educate this group regarding sleep health. It is necessary to mention that the results of this study are primary and need to be confirmed by larger studies.

## Figures and Tables

**Table 1:** Demographic and clinical characteristics of participants.

Variable	*n* (%) or mean (SD)
Age (years)	57.6 (6.02)
Body mass index (kg/m^2^)	26.6 (4.7)
Duration of menopause (years)	6.3 (4.6)
Income (Rials)	668 (45.7) (×10^4^)
Marital status	
Single	58 (15.3%)
Married	322 (84.7%)
ISI*	12.7 (5.1)
ESS**	8.9 (3.7)
OSA***	
Low risk	259 (68.2%)
High risk	121 (31.8%)
Vasomotor domain	3.9 (1.9)
Psychosocial domain	17.4 (7.1)
Physical domain	32.5 (11.9)
Sexual domain	3.1 (1.3)
Total score of MENQOL****	54.5 (19.6)

Data are presented by numbers (percentage) or mean (SD).

*ISI: insomnia severity index.

**ESS: Epworth sleepiness scale.

***OSA: obstructive sleep apnea.

****MENQOL: menopause specific quality of life.

**Table 2:** Multiple regression analysis between demographic and clinical characteristics and EDS, insomnia, and OSA.

	Insomnia			Excessive daytime sleepiness			Obstructive sleep apnea		
	Beta	*P* value	*R* ^2^	Beta	*P* value	*R* ^2^	Beta	*P* value	*R* ^2^
Age	0.62	0.03	0.35	0.004	0.09	0.46	0.04	0.7	0.54
Body mass index	0.53	0.6		0.32	0.01		0.59	0.005	
Duration of menopause	0.71	0.007		0.005	0.4		0.06	0.8	
Income	0.42	0.8		0.88	0.07		0.82	0.09	
Marital status	0.1	0.4		0.52	0.3		0.19	0.1	

**Table 3:** Total score and mean (SD) MENQOL* subscale score in relation to insomnia, EDS**, and OSA***.

	With insomnia	Without insomnia	*P* value	With EDS	Without EDS	*P* value	With OSA	Without OSA	*P* value
	Mean (SD)	Mean (SD)		Mean (SD)	Mean (SD)		Mean (SD)	Mean (SD)	
Total score	62.9 (31.7)	43.5 (21.3)	0.005	74.2 (34.9)	47 (22.7)	<0.001	59.1 (30.1)	53.6 (29.2)	0.21
Vasomotor domain	3.6 (4.01)	1.9 (2.2)	0.002	4.6 (4.3)	2.3 (2.7)	0.006	3.3 (2.8)	2.8 (3.4)	0.25
Psychosocial domain	19.1 (9.03)	14.1 (8.8)	<0.001	19.8 (8.9)	16.02 (9.2)	<0.001	19.7 (8.9)	14.3 (9.2)	0.006
Physical domain	37.02 (20.2)	25.2 (13.2)	0.001	44.2 (21.1)	27.4 (15.2)	<0.001	31.7 (20.5)	32.1 (17.9)	0.87
Sexual domain	3.2 (4.1)	2.9 (3.1)	0.009	5.6 (4.9)	2.03 (2.5)	0.01	3.7 (4.3)	2.9 (3.6)	0.14

*MENQOL: menopause specific quality of life.

**EDS: excessive daytime sleepiness.

***OSA: obstructive sleep apnea.

**Table 4:** Multiple regression analysis between demographic, clinical characteristics, sleep disturbances, and MENQOL* subscales.

	Total score			Vasomotor domain			Psychosocial domain			Physical domain			Sexual domain		
	Beta	*P*	*R* ^2^	Beta	*P*	*R* ^2^	Beta	*P*	*R* ^2^	Beta	*P*	*R* ^2^	Beta	*P*	*R* ^2^
Age	0.609	0.24	0.39	0.44	0.37	0.52	−0.11	0.74	0.37	0.68	0.45	0.49	0.17	0.15	0.53
Body mass index	0.047	0.43		0.62	0.5		−0.71	0.82		0.64	0.6		0.76	0.13	
Duration of menopause	0.27	0.07		0.073	0.51		−0.408	0.081		0.52	0.2		0.055	0.61	
Income	0.205	0.11		−0.32	0.62		0.33	0.82		0.702	0.75		0.13	0.1	
Marital status	−0.16	0.68		0.18	0.41		0.081	0.31		0.43	0.45		−0.61	0.09	
Insomnia	−0.13	0.01		−0.405	0.004		−0.11	0.03		−0.35	<0.001		−0.27	0.008	
EDS**	−0.45	0.03		−0.51	0.02		−0.902	0.003		−0.72	0.007		−0.338	0.04	
OSA***	0.604	0.37		0.17	0.91		−0.407	0.016		0.18	0.28		0.223	0.18	

*MENQOL: menopause specific quality of life.

**EDS: excessive daytime sleepiness.

***OSA: obstructive sleep apnea.
